# Powered knee and ankle prosthesis use with a K2 level ambulator: a case report

**DOI:** 10.3389/fresc.2023.1203545

**Published:** 2023-06-14

**Authors:** Ann M. Simon, Suzanne B. Finucane, Andrea J. Ikeda, R. James Cotton, Levi J. Hargrove

**Affiliations:** ^1^ Center for Bionic Medicine, Shirley Ryan AbilityLab, Chicago, IL, United States; ^2^ Department of Physical Medicine and Rehabilitation, Northwestern University, Chicago, IL, United States; ^3^ Department of Biomedical Engineering, Northwestern University, Evanston, IL, United States

**Keywords:** above-knee amputation, artificial leg, older adult, physical therapy, prosthesis training, rehabilitation, robotic prosthesis, case report

## Abstract

Powered prosthetic knees and ankles have the capability of restoring power to the missing joints and potential to provide increased functional mobility to users. Nearly all development with these advanced prostheses is with individuals who are high functioning community level ambulators even though limited community ambulators may also receive great benefit from these devices. We trained a 70 year old male participant with a unilateral transfemoral amputation to use a powered knee and powered ankle prosthesis. He participated in eight hours of therapist led in-lab training (two hours per week for four weeks). Sessions included static and dynamic balance activities for improved stability and comfort with the powered prosthesis and ambulation training on level ground, inclines, and stairs. Assessments were taken with both the powered prosthesis and his prescribed, passive prosthesis post-training. Outcome measures showed similarities in velocity between devices for level-ground walking and ascending a ramp. During ramp descent, the participant had a slightly faster velocity and more symmetrical stance and step times with the powered prosthesis compared to his prescribed prosthesis. For stairs, he was able to climb with reciprocal stepping for both ascent and descent, a stepping strategy he is unable to do with his prescribed prosthesis. More research with limited community ambulators is necessary to understand if further functional improvements are possible with either additional training, longer accommodation periods, and/or changes in powered prosthesis control strategies

## 1. Introduction

Leg prostheses that actively generate joint torque have been developed to restore ability for individuals with a lower limb amputation. Currently, the Power Knee™ (Ossur, Reykjavik, Iceland), the Intuy Knee (Rebecoon Bionics, Netherlands), and Empower prosthetic foot (Ottobock, Duderstadt, Germany) are the only commercially available powered leg devices. For transfemoral ambulators, control and coordination of a powered knee and powered ankle may allow for improved mobility and function. Currently, all powered leg systems (i.e., powered knee and powered ankle working together) are still in research and development (1–5) and not commercially available to be prescribed to transfemral amputees. Studies report that users can ambulate with more normative or symmetric knee and ankle kinematics and kinetics on level-ground and inclines (4, 6, 7), climb stairs with reciprocal gait (4, 8, 9) and stand up from a seated position with more equal weight distributed between their lower limbs (10, 11).

Nearly all research on powered leg devices is with individuals who are high functioning community ambulators. These individuals, Medicare Functional Classification Level (MFCL) K3 and K4 ambulators (12), are often the first testers of powered prostheses because they ambulate in the community with variable cadence in most environments (13). A subpopulation of these ambulators are also the current intended recipients; US third-party reimbursement of prosthetic components generally limits potential prescription of powered leg devices to individuals categorized at the K3 and K4 level. While they can likely benefit from the joint power these devices provide, individuals with limited community ambulation may have the potential to gain the most benefit.

A systematic review suggested that over half of individuals with transfemoral amputation do not become unlimited community ambulators (14). K2 ambulators, limited community ambulators, are typically prescribed a passive, non-microprocessor controlled prosthetic knee (NMPK). Research has shown the possibility that less advanced prosthetic components may be a barrier to increased community ambulation. When K2 ambulators were provided a passive microprocessor controlled knee (MPK), Ottobock C-Leg or C-Leg Compact, they demonstrated significant improvements in walking speed, ramp and hill negotiation (13, 15, 16). Furthermore, when using a MPK compared to their prescribed NMPK, K2 and K3 ambulators have shown their ability to improve their mobility so as to be evaluated as being at a higher K-level (13, 17) and, demonstrated for K2 level only, significantly decrease uncontrolled falls (13). Even when using MPKs, ambulation with a transfemoral prosthesis remains challenging.

We believe K2 ambulators may gain additional benefit from powered devices but it is relatively unknown how they may respond to the power and the typical increase in weight. An initial case series demonstrated that K2 ambulators could climb stairs with reciprocal stepping strategy using a lightweight powered leg (18). We extend these results to include multiple modes (level walking, inclines, stairs, sit/stand) and discuss modifications to powered leg training protocols (19). Our case study aim was to provide functional mobility training for a powered prosthesis to an individual at the K2 level. Objectives included identifying control system parameters and/or therapist recommended training modifications that needed to be modified based on a lower mobility population and comparing outcomes and preference between the powered prosthesis and the individual's prescribed, passive prosthesis.

## 2. Methods

### 2.1. Participant

The individual was a 70 year old male with a unilateral transfemoral amputation due to blood clot at age 65. He was 1.78 m tall and weighed 88 kg when not wearing his prosthesis. His clinically prescribed passive prosthesis included an Ottobock C-Leg® knee, Ottobock C-Walk foot (Ottobock, Duderstadt, Germany), and an ischial containment socket with flexible inner socket, utilizing skin-fit suction suspension. This prosthesis weighed 4.8 kg (including the socket and pylon connections). He was capable of limited community ambulation typical of a MFCL K2 ambulator and reported no problems using his prosthsesis except occasional low back pain. He used a single-point cane for ambulation, ascended and descended stairs using a step-to strategy, and chose to walk down an incline sideways if no handrail was available. Prior to participation in this study, he had no previous experience walking with a powered prosthesis. This study was approved by the Northwestern University Institutional Review Board and the individual provided written informed consent to participate.

### 2.2. Powered prosthesis fitting and training

**Figure 1:**
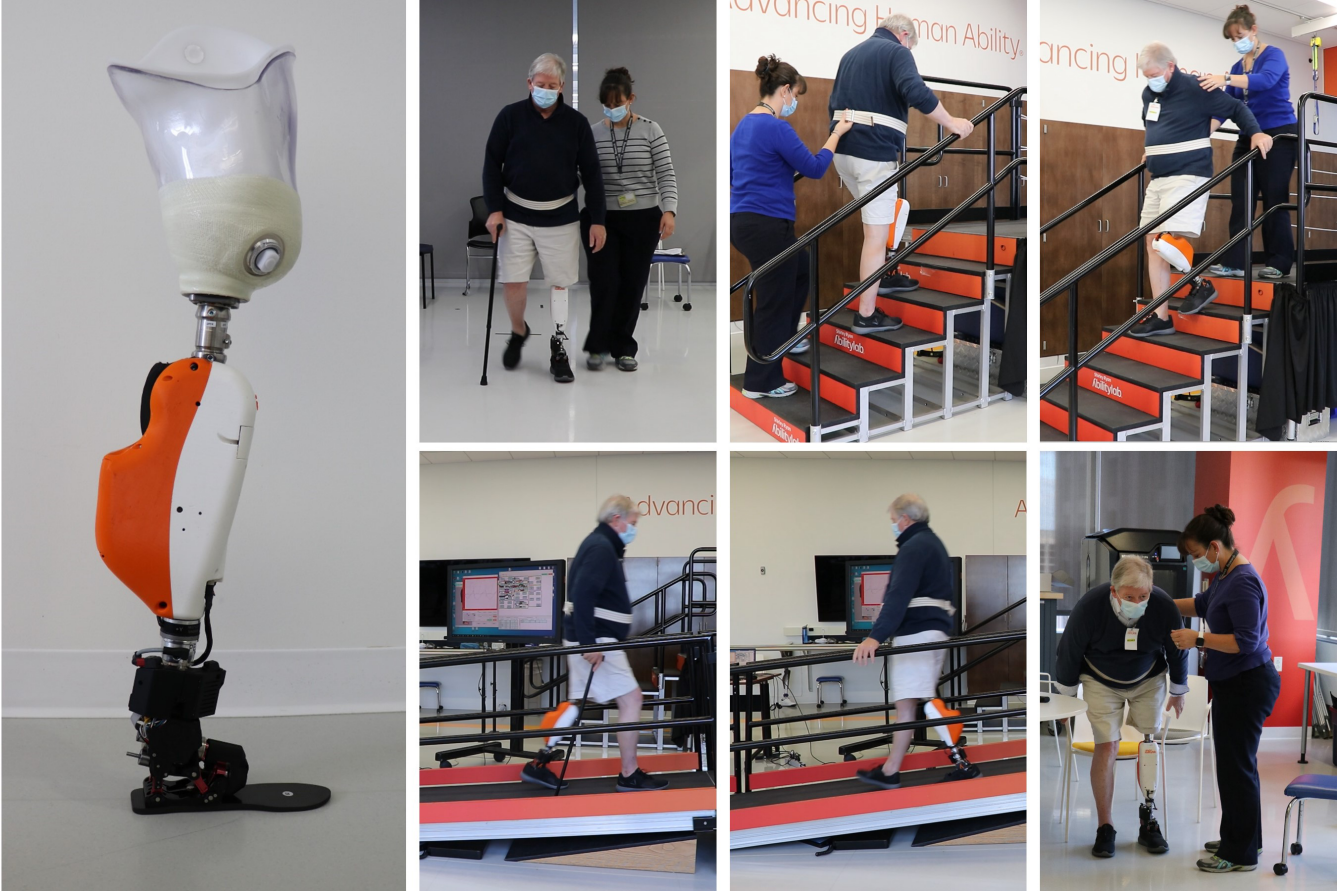
The powered knee and ankle prosthesis and participant training.

A certified prosthetist fit the powered prosthesis. The powered prosthesis included a lightweight powered knee (20), a polycentric powered ankle (21), and a duplicate of the participant's own clinically prescribed socket (Figure 1, *left*), with a total weight of 5.8 kg, including the duplicated socket. Embedded sensors in the prosthesis measured joint position, velocity, and motor currents, a 6 degree-of-freedom load cell, a 6 degree-of-freedom inertial measurement unit, and thigh and shank inclination angles. Prosthetic knee and ankle joint torques were controlled using an impedance-based finite state machine, originally outlined by Goldfarb et al. (6) and further refined for various powered leg prostheses and multiple ambulation modes (3, 7, 10, 22–24). Ambulation modes included standing, level-ground walking, ascending and descending a ramp, ascending and descending stairs, and sit-to-stand/stand-to-sit weight transfers. Similar to previous work, gait phase transitions (e.g., stance to swing) within a mode and ambulation mode transitions between standing, level-ground walking, and sit/stand weight transfers were controlled via thresholds on the embedded sensors (e.g., axial load, thigh inclination angle, sagittal moment). Ambulation mode transitions to and from ramps and stairs were controlled via a key fob (25).

Static and dynamic alignment were assessed and adjusted similar to standard practice. The participant was educated on differences of this device compared to his prescribed prosthesis. Standing and walking training began in parallel bars and progressed to outside the bars with use of his cane and a gait belt. Initial prosthesis parameter adjustment followed previously powered prosthesis literature with community ambulators (K3-K4 level ambulators) (19, 23). Parameters were adjusted to improve gait kinematics and user stability based on both user and clinician feedback with the goal of comfortable and safe ambulation on level ground, inclines, and stairs. Parameters were not specifically optimized to replicate intact-limb kinematics.

The individual returned to participate in eight hours of therapist led in-lab training (two hours per week for four weeks). Training sessions were designed similar to prior powered leg instruction developed with K3/K4 ambulators (19) with additional time focused on static and dynamic balance activities, such as standing and reaching, stepping over objects and manual perturbations to simulate activities of daily living, for improved stability and comfort with the powered prosthesis. Additional planned modifications to accommodate the lower level, K2 participant included (1) progressing slower through training goals with overall less training steps and additional rest breaks to accommodate fatigue, (2) incorporating an assistive device, his cane, for all ambulation modes, (3) increased monitoring of fatigue, back pain, socket comfort, and residual limb condition due to new activities such as reciprocal stair climbing.

Training began with extended time within the parallel bars offering bilateral upper extremity support for standing tasks. Level-ground walking focused on step length, prosthesis stance time and gait speed. Verbal and tactile cues were given to progress from bilateral upper extremity support to use of his cane only. Increased number of standing balance activities were incorporated due to concern for potential loss of balance during all modes. Once comfortable ambulation with the assistive device was achieved, stair climbing and incline walking was introduced. Stair climbing instruction was modified to include minimal assist from clinician due to balance concerns and increased tactile cues for prosthesis foot placement, proper prosthesis loading, and trunk position to achieve comfortable stair ascent reciprocal stepping. Similarly, stair climbing parameters were adjusted for anticipated decreased speed of stair ascent and descent for the K2 level participant. Sit to stand transfers were practiced for all seated rest breaks, with focus on equal weight bearing through both lower extremities and standing balance while reaching for the chair. Verbal and tactile cues for foot and trunk position were needed along with monitoring socket comfort with the increased hip flexion required for stand to sit transitions. We expected that the time frame dedicated to training each ambulation mode would vary based on fatigue or modifications to technique due to potential physical limitations of the participant. Deviations in training and/or parameter settings based on the individual's function and mobility with the powered prosthesis were recorded.

### 2.3. Prosthesis assessment

After training, ambulation was assessed with the powered prosthesis and, in a separate session, with the participant's prescribed prosthesis. Outcome measures performed included the Amputee Mobility Predictor with Prosthesis (AMPPRO) (26), the 10 meter walk test (10 mWT), the Hill Assessment Index (HAI) (27), the Stair Assessment Index (SAI) (28), and the Timed Up and Go (TUG) test. The participant also provided subjective feedback including his preference for and confidence with the device for each ambulation mode.

Lower limb kinematic data were recorded using the Xsens MVN Analyze motion capture system (Xsens Technologies B.V., Enschede, The Netherlands). Seven small wireless motion trackers were secured to the participant's feet, lower legs, and upper legs bilaterally, and the center of the pelvis. The motion trackers were calibrated while standing with feet hip-width apart and during walking. The participant performed three trials each of level-ground walking, ascending and descending a ramp, and ascending and descending a six-step staircase. Kinematic data were exported from the Xsens software and average hip, knee, and ankle angles calculated across gait cycles for each ambulation mode. Additionally, during the level-ground and ramp trials, the participant walked over a GAITRite® Platinum walkway (GAITRite®, Franklin, New Jersey, USA). Temporal-spatial data including speed, step width, stance time, and step length were exported from the GAITRite® CIR 2010 Software. Participant feedback was gathered via a questionnaire which included items to describe the function, ease of use, and cosmesis for both prostheses.

## 3. Results

### 3.1. Powered prosthesis training

The initial fitting and parameter setting session was successful and proceeded similarly to sessions involving K3-K4 level ambulators sessions with the expected difference of slower progress with more rest. Ensuring stability on the powered prosthesis was an important concern for both the participant and clinicians. Walking initiation (i.e., first step after standing that the prosthetic knee actively flexes) and walking termination (i.e., first standing step after continuous walking steps that the prosthetic knee remains extended for standing) needed additional refinement for increased stability. Notably, the participant preferred to initiate walking with his prosthesis side. We modified the powered leg control to only allow walking initiation (i.e., standing to walking transition) when leading with the prosthesis. This change provided the additional stability this participant preferred with step initiation, turning and completing quick starts and stops of gait needed for functional mobility. Another setting change was to reduce knee swing speed (both flexion and extension) for decreased walking speed during all ambulation modes for improved comfort and balance. The participant achieved comfortable stair climbing (ascent and descent) quickly with parameter adjustments to decrease swing speed of the prosthesis. He ambulated over level ground, inclines, and on stairs with reciprocal stepping during the first session.

Incline ambulation required additional instruction time compared to higher level amputees who completed training with the powered prosthesis. This participant reported difficulty with incline walking with his clinically prescribed prosthesis, stating discomfort and uncertainty, specifically during ramp descent. During the first attempt of ramp descent, verbal and manual cues were required for the participant to load the powered prosthesis for stance stability. After the participant completed two steps down the ramp, he felt the stance stability of the powered prosthesis and advanced forward over the foot to initiate swing. The participant advanced quickly through incline walking resulting in the ability to ascend and descend the ramp with his single point cane and one handrail. Throughout incline walking training, the participant expressed his comfort with ramp descent and confidence with the stance support of the powered prosthesis as compared to his clinically prescribed prosthesis.

Upon completion of the training sessions, the participant demonstrated improved standing balance, and decreased therapist support for all ambulation modes (Figure 1). The participant continued to use his single point cane for level-ground walking with contact guard/stand by assist from the clinician. The participant displayed improved stability and confidence with all mobility tasks, this was demonstrated by progressing from use of bilateral handrails for incline walking, to use of one handrail and single point cane for both ascent and descent of the ADA compliant ramp. During stair climbing, the participant progressed from needing bilateral handrails, minimal assist and verbal cues for prosthesis placement to requiring supervision with bilateral hand rails, along with consistent prosthesis placement and variable speed during stair climbing with confidence. The participant consistently completed sit to stand transfers with supervision to a standard height chair with arm rests.

The participant adhered to and tolerated the training protocol schedule (two hours per week for four weeks) without issue. There were no unanticipated or adverse events that occurred during training nor during assessment. There was no additional follow up after the post-training assessment of the powered prosthesis or his prescribed, passive prosthesis.

### 3.2. Prosthesis assessment

**Figure 2:**
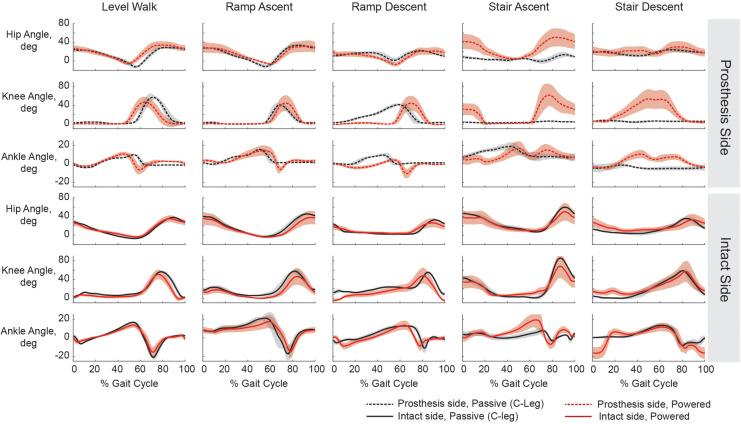
Joint kinematics for level-ground walking, ramp ascent and descent, and stair ascent and descent while using the participant's prescribed prosthesis (black) and the powered prosthesis (red). Hip, knee, and ankle kinematics are plotted for both the prosthesis side (dashed) and intact sides (solid). The participant used his single point cane for level walking, ramp ascent, and ramp descent with both devices. On stairs with his prescribed prosthesis he used a step-to strategy, maintaining a straight prosthetic leg both for ascent and descent. On stairs with the powered prosthesis he used a reciprocal strategy and bilateral handrail support. Shaded regions represent +/−1 standard deviation.

Level-ground walking velocity was similar between devices; although the 10mWT showed slightly slower velocity with the powered prosthesis compared to the prescribed prosthesis (Table 1), GAITRite® trials showed the opposite result (Table 2). Spatio-temporal results were similar between devices as well (Table 2). During walking, the powered prosthesis provided powered knee swing and late-stance plantarflexion but did not alter intact-side kinematics (Figure 2).

**Table 1:** Outcome measures.

Device	10 mWT, m/s	AMPPRO	SAI		HAI		TUG, sec
			Ascent	Descent	Ascent	Descent	
Prescribed Prosthesis	0.72	32	3	3	6	6	22.3
Powered Prosthesis	0.64	33	11	11	7	6	28.0

AMPPRO: score range 0–47; higher score = better mobility.

SAI: ordinal scale of 0–13 where 0 = Cannot do, and 13 = Step over step without rail or assistive device.

HAI: ordinal scale of 0–11 where 0 = Cannot do, and 11 = Forward, even step, without assistive device.

**Table 2:** Temporal-spatial data recorded during level-ground and incline walking.

Mode	Device	Velocity, m/sec	Step Width, m	Step Length, m		Step Time, sec		Swing Time, sec		Stance Time, sec	
				Prosthesis	Intact	Prosthesis	Intact	Prosthesis	Intact	Prosthesis	Intact
Level Walk	Prescribed	0.60 (0.05)	0.17 (0.02)	0.53 (0.04)	0.50 (0.03)	0.87 (0.07)	0.86 (0.07)	0.64 (0.11)	0.50 (0.05)	1.10 (0.07)	1.22 (0.11)
	Powered	0.66 (0.06)	0.16 (0.03)	0.53 (0.06)	0.54 (0.03)	0.84 (0.08)	0.76 (0.03)	0.63 (0.07)	0.41 (0.03)	0.97 (0.05)	1.20 (0.10)
Ramp Ascent	Prescribed	0.62 (0.06)	0.20 (0.03)	0.55 (0.04)	0.59 (0.05)	0.93 (0.06)	0.90 (0.06)	0.70 (0.07)	0.52 (0.12)	1.13 (0.07)	1.31 (0.17)
	Powered	0.55 (0.05)	0.16 (0.01)	0.52 (0.03)	0.59 (0.02)	1.07 (0.07)	0.94 (0.04)	0.74 (0.06)	0.49 (0.05)	1.29 (0.10)	1.54 (0.09)
Ramp Descent	Prescribed	0.34 (0.04)	0.16 (0.02)	0.37 (0.04)	0.37 (0.06)	1.26 (0.18)	0.95 (0.05)	0.86 (0.11)	0.41 (0.02)	1.34 (0.26)	1.81 (0.16)
	Powered	0.43 (0.03)	0.14 (0.02)	0.38 (0.02)	0.46 (0.03)	1.03 (0.05)	0.94 (0.04)	0.77 (0.06)	0.45 (0.02)	1.20 (0.03)	1.51 (0.09)

The largest difference in functional assessment was seen during stair climbing with the powered prosthesis compared to his prescribed prosthesis (SAI, Table 1). This increase was due to his ability to climb with reciprocal stepping for ascent and descent (Figure 2).

While ascending the ramp, minimal spatio-temporal and intact side kinematic differences were seen between devices (Table 2, Figure 2). While descending the ramp, the participant preferred to keep powered knee extended during stance and not allow it to flex until just before toe off, but with his prescribed prosthesis, he let it flex more gradually and earlier in stance. (Figure 2). The participant had a slightly faster velocity and more symmetrical stance and step times with the powered prosthesis compared to with his prescribed prosthesis (Table 2).

### 3.3. Participant feedback

The participant was satisfied with his prescribed prosthesis on all surveyed items (donning, level-ground walking, incline walking, stair climbing, cosmesis, and weight of prosthesis) except for physical exertion while walking on both level-ground and inclines. Reasons listed for dissatisfaction included fatigue and decreased stability when walking up or down inclines. The participant stated that he avoids ramps or uneven terrain in the community due to instability and level of exertion required to complete these tasks. Even though the participant has been instructed on use of his prescribed prosthesis to descend stairs in a reciprocal pattern, he reported decreased stability and confidence due to the lack of support provided by the passive prosthetic knee. Therefore, he chooses to use a step-to pattern during stair ascent and descent.

Throughout the powered prosthesis training sessions, the participant reported increased ease of walking due to increased comfort and confidence in the powered stance stability. He reported higher satisfaction with incline walking and stair climbing and reported less physical exertion experienced while walking as compared to his prescribed prosthesis. He was less satisfied with the powered prosthesis compared to his passive prosthesis for the transition from standing to sitting, cosmesis, and weight of the prosthesis. The participant stated that he felt safer when walking on inclines with the powered prosthesis due to the stability of the device. When asked which device he would prefer to use for each activity, he selected the powered prosthesis over his prescribed prosthesis for level-ground walking, incline walking, stair climbing and sit to stand transfers (i.e., all surveyed activities). Even though he stated dissatisfaction with the weight and cosmesis of the powered prosthesis, he stated he would not want to trade functionality for improved cosmesis.

## 4. Discussion

Functional mobility training with the powered knee and ankle prosthesis was successful with this K2 ambulator. Overall, his training largely mirrored training K3 ambulators on a powered device, with more frequent and longer duration breaks due to fatigue resulting in less total training steps per session. The participant demonstrated increased confidence and decreased therapist support as training progressed within each session, but only moderate transfer to the start of the next training or assessment session (scheduled weekly due to his availability). While powered devices may allow these individuals to do tasks they are unable to do with their passive device (e.g., climb stairs with a reciprocal gait), learning those tasks may still be challenging due to non-prosthetic related limitations such as decreased fitness levels, decreased strength, and balance stability.

During assessment, while the participant showed no noteworthy temporal-spatial or kinematic changes for level-ground walking, it is encouraging that the added weight and complexity of the powered prosthesis did not cause a detriment to this activity. For stair ambulation with the powered prosthesis there was a quantifiable benefit, an increase of eight points on the stair assessment index, for both ascent and descent. The increase in score was attributed to his ability to now ascend and descend stairs with reciprocal stepping using both handrails. Reciprocal stair ascent was easy for him to learn and is only possible due to the active knee power. Active ankle dorsiflexion allowed for proper upper body positioning prior to active knee extension to climb the stair. Kinematics of stair ascent (Figure 2) show a pause between prosthetic knee full extension and subsequent swing. The participant may be waiting for confirmation that the prosthetic knee is fully straight prior to initiating stair stepping of the intact side foot; it is unclear whether additional training could improve this timing of stair weight transfers. While some individuals with a transfemoral amputation can descend stairs using a reciprocal gait with a passive prosthesis, our participant cannot, as is typical of K2 ambulators. He is unable to control the rate of descent using his residual limb musculature resulting in instability as the the prosthetic knee flexes too fast for him to compensate. With the powered prosthesis, the rate of descent was controlled using active knee power, thereby providing him stability throughout the whole movement. Transitioning to a reciprocal gait on stairs could have the implication of reducing sound side overuse injuries over the long-term.

Additionally, during incline walking the participant similarly noted increased confidence on the powered prosthesis. Increased confidence and stability on a prosthesis are not negligible outcomes; more than half of adults with lower limb amputations fall at least once a year (29) and concern for falling generally impacts their quality of life (30). No noteworthy quantitative differences were seen for ramp ascent. For ramp descent the participant preferred the powered prosthesis remained extended and supportive during stance, commenting that the way he had to “ride” the knee down with his prescribed prosthesis felt unstable and if handrails weren't available he would have chosen to go down the ramp side stepping so that the knee wouldn't bend. With the powered prosthesis, similar to stair descent, the rate of descent was controlled. It is possible that the additional range of motion of the ankle provided a longer duration of foot flat on the ramp contributing to his ability to walk down the ramp with more symmetrical stance step times. While the one-point improvement on the AMPPRO is minimal, and may improve with additional training, this is the first time demonstrating successful use of this metric with a powered prosthesis.

The self-reported user experience was overall positive with the powered prosthesis. Although there were no measurable differences for level-ground walking, he did prefer to use the powered prosthesis over his prosthesis for this activity. Upon completion of incline walking instruction and training, the participant reported confidence in the stability of the powered prosthesis and prefered its use over his prescribed prosthesis for uneven terrain. This was demonstrated by his ability to ambulate up an incline without handrails, only and using his straight cane for support. With his prescribed prosthesis, he had reported falls or loss of balance when the prosthetic knee unexpectently flexes or “gives way”, especially when walking on a declined surface. Due to the active stance stability of the powered prosthesis, the participant reported ease of use also when turning or stepping in small spaces for improved functional mobility. He was less satisfied with the powered prosthesis for the transition from standing to sitting, potentially due to increased resistance into flexion for a seated position. Use of this feature may improve with increased training and acclimation time.

The powered leg control did not need many modifications beyond what has been typically configured (23). The change to only allow walking initiation when leading with the prosthesis is potentially not necessary for all K2 users but did provide additional stability for this individual. While the powered prosthesis allowed variable walking speed (31), knee swing speed was sometimes too fast and occasionally caused balance issues. Upon further inspection, this is not surprising since his walking speeds (level-ground: ∼0.7 m/s; ramp ascent 0.55 m/s; ramp descent 0.43 m/s) were at or below the range of speeds previously recorded as slow for K3/K4 ambulators (level-ground: 0.85 to 1.2 m/s; ascent: 0.7 to 1.0 m/s) (31, 32). Further reduction of knee swing speed across all modes resulted in better timing for his preferred speed. Although no additional adjustments were made for stair climbing that weren't normally modified for higher functioning ambulators (9, 23), joint power during stair climbing and sit-to-stand movements likely could have been better optimized if time had allowed (33). It is possible that with further training and configuration, TUG times could decrease with the powered prosthesis.

While this is a case study and more research with K2 ambulators using powered devices is necessary to see if similar results extend to a larger K2 ambulator population with a transfemoral amputation, it is an important first step in working towards that goal. Other limitations include that pre-training assessments could provide further insight into how fast or slow K2 ambulators accommodate to various activities within the training protocol and that the level of training received for the participants prescribed prosthesis was unmeasured.

Evidence has shown that providing K2 ambulators with a microprocessor-controlled knee could improve their functional mobility (13, 34); the potential of these same ambulators with a powered knee and ankle prosthesis is currently unknown with the larger population. While further functional improvements may be possible, increased accommodation time and/or frequency of training visits may be necessary. Innovation of control strategies may be necessary for K2 ambulators to better utilize the power these powered prostheses provide. Finally, since increased weight of the powered components may effect ambulation, it is also possible that K2 ambulators may benefit from a lighter weight device with power at only one joint (e.g., knee or ankle) as opposed to both as reported in this case report.

## Data Availability

The raw data supporting the conclusions of this article will be made available by the authors, without undue reservation.
