# Corrigendum: Powered knee and ankle prosthesis use with a K2 level ambulator: a case report

**DOI:** 10.3389/fresc.2023.1351558

**Published:** 2023-12-21

**Authors:** Ann M. Simon, Suzanne B. Finucane, Andrea J. Ikeda, R. James Cotton, Levi J. Hargrove

**Affiliations:** ^1^ Center for Bionic Medicine, Shirley Ryan AbilityLab, Chicago, IL, United States; ^2^ Department of Physical Medicine and Rehabilitation, Northwestern University, Chicago, IL, United States; ^3^ Department of Biomedical Engineering, Northwestern University, Evanston, IL, United States

**Keywords:** above-knee amputation, artificial leg, older adult, physical therapy, prosthesis training, rehabilitation, robotic prosthesis, case report

A Corrigendum on Powered knee and ankle prosthesis use with a K2 level ambulator: a case report By Simon AM, Finucane SB, Ikeda AJ, Cotton RJ and Hargrove LJ (2023). Front. Rehabil. Sci. 4:1203545. doi: 10.3389/fresc.2023.1203545

**Incorrect Funding**

In the published article, there was typo in the Funding statement. One funding agency had a typo stating incorrectly ‘MLS Renewed Hope Foundation’ instead of ‘MSL Renewed Hope Foundation’. The full correct Funding statement appears below.

